# Using mDixon to remove motion artifacts in carotid artery vessel wall MRI

**DOI:** 10.1186/1532-429X-14-S1-P273

**Published:** 2012-02-01

**Authors:** Jinnan Wang, Peter Koken, Niranjan Balu, Chun Yuan, Peter Boernert

**Affiliations:** 1 Philips Research, Seattle, WA, USA; 2 Philips Research Europe, Hamburg, Germany; 3 Radiology, University of Washington, Seattle, WA, USA

## Background

Black blood carotid artery wall imaging can evaluate not just atherosclerotic plaque burden but also high risk components like lipid core and intraplaque hemorrhage. A current limitation in its clinical application, however, is its susceptibility to motion artifacts caused by involuntary motions like swallowing, coughing and breathing. Due to the natural bright signal from subcutaneous fat, unsuppressed fat signal contributes to the majority of motion artifacts in the carotid artery wall region. The fat signal cannot always be properly suppressed using spectrally selective RF pulses due to magnetic field inhomogeneities. Modified Dixon (mDixon) technique, however, is less susceptible to field inhomogeneities because it can separate fat signal without relying on the absolute frequency of the fat spectral.

The aim of this study is to explore the feasibility of using mDixon techniques to better separate the strong fat signal and thus reduce motion artifacts near carotid wall region.

## Methods

A two-point mDixon acquisition was programmed to make an automatic extra acquisition with the acquisition window shift after each regular TSE acquisition. The time shift was selected to be 1.15ms, which corresponds to exactly 180 phase separation between water and fat signal at 3T. All images were acquired using a whole body scanner (Philips Achieva, R3.21, the Netherlands) with a dedicated carotid coil. 5 patients with diagnosed carotid atherosclerosis diseases were scanned with both regular spectral selective fat saturationT1 MSDE and an mDixon T1 MSDE sequences. After each mDixon acquisition, the water image was automatically calculated by the algorithm reported before (1). The image quality of the carotid artery on each image was individually evaluated according to an established method (2).

## Results

**Figure 1:**
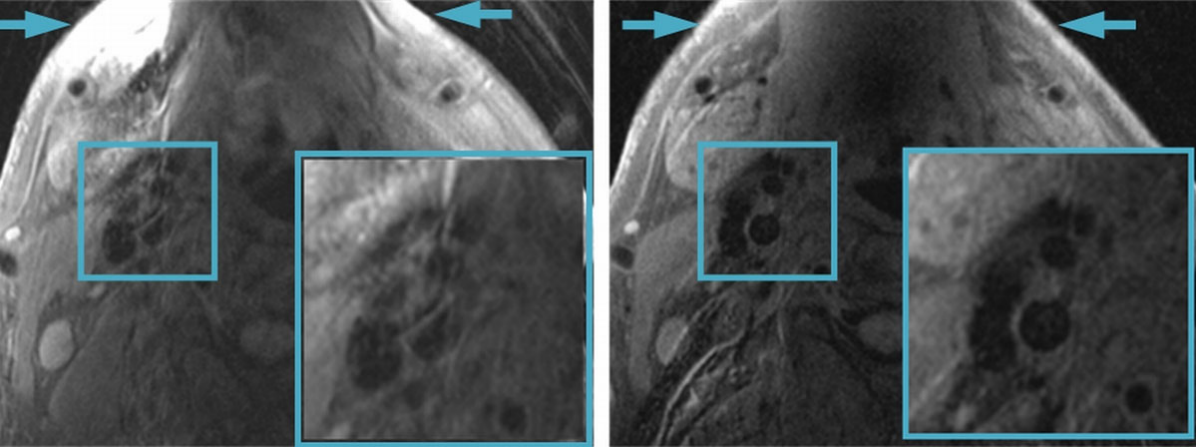
Image quality comparison between spectrally selective fat saturation image (left) and water image from mDixon (right). Images were acquired on the same subject. As the arrows indicated, the peripheral fat saturation is dramatically improved on mDixon images. The vessel wall delineation has also improved due to the significant reduction of motion artifacts from fat (zoomed images).

The black blood mDixon image presented significantly improved fat saturation at the peripheral regions of the neck (Fig.1 arrows) on all subjects. On one subject with significant motion artifacts, mDixon showed remarkable image quality improvement around the carotid artery region. It is noteworthy that although motion artifacts can still be observed on mDixon images, its reduced brightness helped improve the delineation of the carotid arteries.

## Conclusions

mDixon water fat separation technique improved fat suppression in carotid black blood MRI and helped reduce motion artifacts from fat in carotid artery wall images. Benefited from the improved fat suppression, dramatically improved carotid wall delineation can be observed when motion artifact presents.

## Funding

N.A.

